# The Application of Flame Spread Theory to Predict Material Performance

**DOI:** 10.6028/jres.093.007

**Published:** 1988-02-01

**Authors:** J. G. Quintiere

**Affiliations:** National Bureau of Standards, Gaithersburg, MD 20899

**Keywords:** flame spread, heat transfer, ignition, material properties, small scale fire tests, thermal radiation

## Abstract

A review is presented of recent work which attempts to apply flame spread theories to a wide range of materials. The approach is based on using the theories to develop correlations from material data. The data are derived from small scale tests and are expressed in terms of “properties.” Various radiant heating apparatus are discussed, and a wide range of results are presented. The focus of the application is fire spread on walls.

## Introduction

There are many different tests for assessing the flammability of materials. In most cases these tests are for the purpose of evaluating interior finish materials and products, particularly wall and ceiling applications. In general, all of the tests express their results in terms of some observations or measurements. These are then used to derive a relative ranking scale on which to evaluate materials. Unfortunately, the bases of these ranking scales are arbitrary, and therefore results from one test do not necessarily agree with another. Each test measures some combination or aspect of flammability; namely, ignition, flame spread, and energy release. But none attempts to relate their measured test results to theories of ignition, spread or combustion. Consequently, the test results are limited in their use, but often widely applied.

This practice is in sharp contrast to the evaluation of material performance in other fields. If we are interested in the heat transfer characteristics of materials, we seek their thermal properties. If we are interested in the strength of materials, we would seek their modulus of elasticity and yield stress. Then we would seek to understand how the material is to be used and analyze that configuration based on the principles of heat transfer or structural analysis. If the materials were complex in form, we would expect the property data to be “effective” since the engineering analysis we would use would most likely be based on a model for simple homogeneous materials. For example, a measured thermal conductivity of a foam material would represent all the underlying heat transfer processes in the foam. Its measured thermal conductivity would not be that of the pure material or the entrapped air in the foamed material, but it would be an “effective” value based on Fourier’s law of heat conduction applied to the foam. Obviously, the effects on a material in a fire are more complex than this. But by using simple theories based on scientific analysis, we should be able to derive and utilize “effective” property data in an analogous fashion.

In this presentation, “simple” theories for ignition and flame spread will be examined in order to basis for analyzing test results for the materials. Experimental correlations for a particular ignition or flame spread process, when based on theory, would then yield values for the parameters of the theory. As long as these parameters remain reasonably constant over an appropriate range of conditions, or correspond to true material properties ideally, we can consider them to be “effective” property values for the processes of ignition and flame spread. In this way, we seek to develop test procedures to measure “effective material fire properties.” Furthermore, when combined with theory, these properties can be used over a wide range of fire conditions for predicting aspects of ignition and flame spread.

The principal focus for much of the work has been on fire spread over wall materials. Hence the materials are vertically oriented. The relationship to ignition and spread on horizontal materials will be examined under limited circumstances. Only piloted ignition was considered since it will be shown to bear directly on flame spread. Lateral (or horizontal) flame spread data were considered under conditions of natural convection for many materials under a wide range of radiant heating. For some materials, results were compared to downward flame spread under the same heating conditions. A special apparatus was used to measure flame height and heat transfer relevant to upward flame spread. These results were then used to examine aspects of upward flame spread on two different materials—polymethylmethacrylate (PMMA) and Douglas fir particle board.

## Theoretical Aspects

A theoretical basis is sought that is simple in application, yet sufficient for predicting a material’s performance. The theory should be consistent with more fundamental analyses. We could turn directly to results in the literature on fundamental aspects of flame spread, but these generally ignore the effects of external heating. Since most materials require external heating (under normal oxygen conditions) to enable flame spread, this will be a necessary part of a test to derive flame spread data.

**Figure 1:**
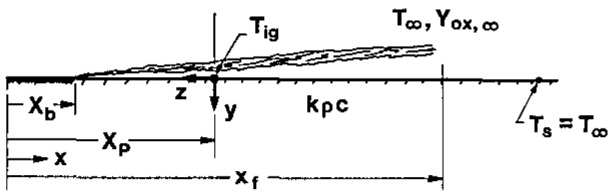
Model for flame spread.

Without a loss in generality of the end results, a thermal theory of flame spread (and ignition) will be considered. The diagram in figure 1 shows a flame spreading over the surface in the *x*-direction. The flame is depicted as if it is being blown by a wind. The fuel is burned out over region *x*_b_, pyrolyzing over region *x*_p_−*x*_b_, and no degradation or vaporization occurs for *x* >*x*_p_. The tip of the flame is given by *x*_f_ and the surface ahead of the pyrolyzing region receives most of the flame heat transfer over this region (*x*_f_−*x*_p_). The initial temperature is given by *T_∞_* and the pyrolysis front *x*_p_ is defined by the position where the temperature achieves the ignition temperature (*T*_ig_) on the surface.

For steady flame spread without external heating it is usually common to define a new coordinate system moving with *x*_p_. With external heat transfer it is better to consider a fixed position *x* and only onedimensional conduction in the *y*-direction. The latter approach will be taken. Also we will consider that the heat flux from the flame ${\dot{q}}_{f}^{\prime \prime }$ is constant and uniform over the time period Δ*t* before *x*_p_ reaches the position *x*; and that the flame spread velocity *V*_p_=d*x*_p_/d*t* is also constant in this period. Furthermore, Δ*t* is defined to be equal to (*x*_f_−*x_p_*)*/V*_p_ where the flame heat flux is applied only over the region *x*_f_−*x*_p_. It will turn out that the general form of the result derived under these conditions will be equivalent with analyses for both wind-aided or opposed flow flame spread conditions. The distinction will lie in how we interpret ${\dot{q}}_{f}^{\prime \prime }$ and *x*_f_−*x*_p_. Also since it will turn out that *V*_p_ will generally vary with time, the assumptions just imposed can be regarded as quasisteady in that at any position *x* = *x*_p_, the velocity is considered constant in its vicinity.

Let us consider the case of a semi-infinite solid. This will result in the thermally-thick case which can be shown to apply to most common organic solids (*k/ρc*~O(10^−3^)cm^2^/s) in applications of construction. This follows since the depth of heating due to the flame (or for conditions of ignition) would be O(2) to O(5)mm so that materials of thicknesses greater than this, or of a combined thickness with a substrate material greater than this, would apply to the semi-infinite result. The problem can then be posed as follows:

$$\begin{array}{l} \text{at}\;\text{position}\;x,\\ \;y=0,-k\frac{\partial T}{\partial y}={\dot{q}}^{\prime \prime }\left(x,t\right)+h\left(T-T_{\infty }\right) \end{array}$$ (1)

$$y\to \infty ,\;\;T=T_{\infty }$$ (2)

$$t=0,\;\;T=T_{\infty }$$ (3)

where

$${\dot{q}}^{\prime \prime }\left(x,t\right)={\dot{q}}_{f}^{\prime \prime }+{\dot{q}}_{e}^{\prime \prime }.$$ (4)

The flame heat flux is defined as described above, and ${\dot{q}}_{e}^{\prime \prime }$ can be a function of *x* and *t*. The governing equation is given as

$$\frac{\partial T}{\partial t}=\frac{k}{\rho c}\frac{\partial^{2}T}{\partial y^{2}},$$ (5)

where *k, ρ*, and *c* are the conductivity, density and specific heat, respectively. This can be solved for the stated conditions. The heat loss, although expressed in a linearized form where the coefficient *h* is constant can be thought of physically as a radiative surface loss under wind-aided conditions or both a radiative and convective loss under opposed flow conditions. A solution to this problem was developed previously [1]. By examining the solution for the surface temperature at *y* = 0 and imposing the condition that the pyrolysis front reaches *x* (i.e., *x* = *x*_p_) when *T = T*_ig_, it can be shown that

$$\begin{array}{c} T_{\mathrm{ig}}-T_{\infty }=\frac{{\dot{q}}_{f}^{\prime \prime }}{h}\;\left(1-\exp\;\beta \;\mathrm{erfc}\;\sqrt{\beta }\right)\\ \;+\int_{0}^{\tau }\frac{{\dot{q}}_{e}^{\prime \prime }\;\left(x_{p},\tau^{\prime }\right)}{h}\;[\frac{1}{\sqrt{\pi \left(\tau -\tau^{\prime }\right)}}\\ \;-\exp\left(\tau -\tau^{\prime }\right)\mathrm{erfc}\sqrt{\left(\tau -\tau^{\prime }\right)}]d\tau^{\prime }, \end{array}$$ (6)

where

$$\tau =\frac{h^{2}t}{k\rho c},$$ (7)

and

$$\beta =\frac{h^{2}}{k\rho c}\frac{\left(x_{f}-x_{p}\right)}{V_{p}}.$$ (8)

This result implies that the required temperature rise for flame spread at *x* = *x*_p_ needs to be composed of a contribution from the flame $\left({\dot{q}}_{f}^{\prime \prime }\right)$ and a contribution from the surroundings $\left({\dot{q}}_{e}^{\prime \prime }\right)$. For the special case where ${\dot{q}}_{e}^{\prime \prime }$ is only a function of *x*, and where *β* is small, or equivalently heat losses are ignored or are unimportant with respect to the flame heating component, it can be shown that

$$\begin{array}{c} T_{\mathrm{ig}}-T_{\infty }=\frac{2\;{\dot{q}}_{f}^{\prime \prime }\sqrt{\left(x_{f}-x_{p}\right)}}{\sqrt{\pi \;k\;\rho c\;V_{p}}}\\ \;+\frac{{\dot{q}}_{e}^{\prime \prime }\left(x_{p}\right)}{h}\;\left(1-\exp\;\left(\tau \right)\;\mathrm{erfc}\;\sqrt{\tau }\right). \end{array}$$ (9)

This will be the governing equation for flame spread. It can be rearranged to express *V*_p_ explicitly as follows

$$V_{p}=\frac{{\left({\dot{q}}_{f}^{\prime \prime }\right)}^{2}\;\left(x_{f}-x_{p}\right)}{\frac{\pi }{4}k\rho c\;{\left(T_{\mathrm{ig}}-T_{s}\right)}^{2}},$$ (10)

where

$$T_{s}-T_{\infty }\equiv \frac{{\dot{q}}_{s}^{\prime \prime }\left(x_{p}\right)}{h}\;\left[1-\exp\left(\tau \right)\mathrm{erfc}\;\sqrt{\tau }\right],$$ (11)

or *T*_s_−*T_∞_* is the temperature rise due to the external heating.

For the case of pure ignition with a pilot that serves to ignite the flammable mixture, but imparts no heat to the solid itself, a thermal model would yield similar results. Indeed, it follows directly from eq (11) that for radiative ignition

$$T_{\mathrm{ig}}-T_{\infty }=\frac{{\dot{q}}_{e}^{\prime \prime }}{h}\;\left[1-\exp\left(\tau \right)\mathrm{erfc}\;\sqrt{\tau }\right],$$ (12)

where the minimum heat flux for ignition would be ${\dot{q}}_{0,\mathrm{ig}}^{\prime \prime }\equiv h\;\left(T_{\mathrm{ig}}-T_{\infty }\right)$.

These results provide a framework for identifying specific ingredients one needs in order to predict flame spread or radiative ignition for materials. From eq (10) it is seen that the material “properties” *kρc* and *T*_ig_ need to be determined. The quantity *kρc* may be available for some materials, but it will depend on temperature and consequently Values at normal atmospheric temperature will not directly apply. Moreover, it should be noted that both *k* and *c* tend to increase generally for solids with temperature. Also *kρc* expresses the heat loss aspect of the solid and the thermal model ignores any pyrolysis effect, so that *kρc* as used here must also represent some heat loss due to pyrolysis. Hence *kρc* is an effective property.

The ignition temperature represents the surface temperature required to produce a flammable mixture just at the lower flammable limit for the flow and flame or ignition conditions under consideration. Under similar flow (or mixing) conditions we would expect the ignition temperature to be roughly a constant for a given material over the range of heating conditions relevant here of roughly 1 to 6 W/cm^2^. Although it is difficult to measure the surface temperature at ignition, it is possible to infer an (effective) ignition temperature by determining experimentally the critical radiative heat flux for piloted ignition, i.e.,

$${\dot{q}}_{0,\mathrm{ig}}^{\prime \prime }=h_{c}(T_{\mathrm{ig}}-T_{\infty })+\sigma ({T_{\mathrm{ig}}}^{4}-{T_{\infty }}^{4})\equiv h\;(T_{\mathrm{ig}}-T_{\infty }),$$ (13)

where here a black surface has been assumed, and the convective coefficient *h*_c_ explicitly, represents the effect of the specific flow or orientation conditions of the process. An ignition temperature as determined in this way is the one required by the thermal models given above for both ignition and flame spread.

Finally, the parameters ${\dot{q}}_{f}^{\prime \prime }$ and *x*_r_−*x*_p_ represent the flame heat flux and flame length (extension beyond the pyrolysis zone). These are not properties in the obvious sense. We need to examine these more closely for specific cases of flame spread.

## Ignition

**Figure 2:**
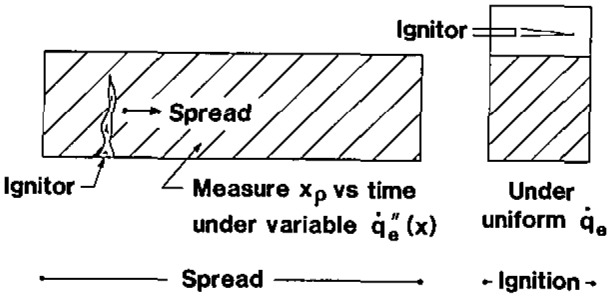
Schematic of apparatus to measure ignition and lateral flame spread.

We have utilized a device [2,3] which imposes a constant and uniform heat flux over a vertically mounted sample. The sample is backed by an inert insulating material and a pilot flame acts above the sample adjacent to a contiguous wall. This is shown in figure 2. The sample face is approximately 10 × 15 cm high.

By conducting experiments at various levels of ${\dot{q}}_{e}^{\prime \prime }$ the times to ignite can be determined. Also, by bracketing, the critical ignition flux can be determined. It has been found that for many materials [2,3], these experimental results can be correlated by the following relationship:

$$\frac{{\dot{q}}_{0,\mathrm{ig}}^{\prime \prime }}{{\dot{q}}_{e}^{\prime \prime }}=F\left(t\right)=\left(\begin{array}{r} b\sqrt{t},t⩽t^{*}\\ 1,t⩾t^{*} \end{array}\right).$$ (14)

The function *F*(*t*) is the empirically determined counterpart to $\left[1-\exp\left(\tau \right)\mathrm{erfc}\;\sqrt{\tau }\right]$ of eqs (11) and (12). Since eqs (11) and (12) were the result of a solution based on linearized heat loss, *F*(*t*) should be considered the result for the actual heat loss experienced with the non-linear radiative loss especially. It has been surprising but fortuitous that the simple functional form of eq (14) has been satisfactory in many varied and complex materials. As *t* or *τ* → ∞, $1-\exp\left(\tau \right)\mathrm{erfc}\;\sqrt{\tau }$ approaches 1 so that t* in *F*(*t*) can be regarded as a time to reach equilibrium or steady state in the material. Also as *t* or *τ*→0, $1-\exp\left(\tau \right)\mathrm{erfc}\;\sqrt{\tau }$ approaches $2h\sqrt{t}/\sqrt{\pi k\rho c}$. Hence since *F*(*t*) follows this behavior for *t* ⩽t*, it follows that

$$b=\frac{2h}{\sqrt{\pi k\rho c}}.$$ (15)

Thus, *kρc* can be derived. The ignition data analysis yields the two effective properties, *kρc* and *T*_ig_, for a material. These should be applicable for both ignition and flame spread.

**Figure 4:**
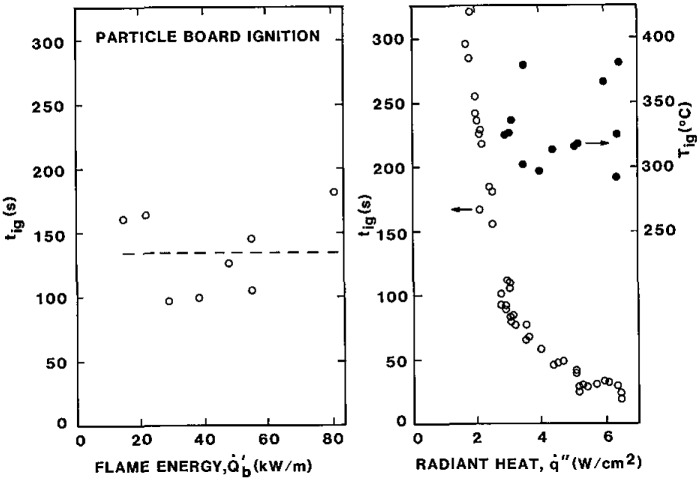
Flame and piloted radiative ignition of Douglas fir particle board in a vertical orientation.

Some examples of results [3] are shown in figures 3a and 3b. These results were obtained in a vertical orientation in a device for which *h*_c_ was determined to be 15 W/m^2^-K under conditions of natural convection [2,3]. From eq (13) the derived ignition temperature for the polycarbonate sample (fig. 3a) was found to be 528 °C and for the carpet of figure 3b a value of 412 °C was determined. Of course, accuracy cannot be assured to three significant figures. Our experience has ranged from values as low as 280 °C for a form of polymethylmethacrylate (PMMA) to 620 °C for a fire retarded plywood. Most materials seem to fall in a range of 350 to 450 °C. Comparison with measured values of surface temperature have been done on a limited basis with encouraging results. The measurement technique used has been described by Atreya et al. [4]. Figure 4 shows results for ignition in a vertical orientation for Douglas fir particle board. This illustrates typical results under direct flame heating $\left({\dot{Q}}_{b}^{\prime }\right)$ from a line burner diffusion flame at the base of the sample as well as by radiant heating with a pilot flame.

It should be mentioned that the wood shown here and all other samples were tested under laboratory temperature and humidity that remained fairly constant at 20 °C and 50% RH so that changes in results due to wide variations in moisture content have been minimized. Table 1 summarizes how these measured T*_ig_* values compare with the inferred *T*_ig_ as derived from ${\dot{q}}_{0,\mathrm{ig}}^{\prime \prime }$ and eq (13). In figure 4 the surface temperatures measured at a fixed radiant heat flux have been plotted. Results are also shown for PMMA determined similarly.

The corresponding derived values for *kρc* compared to values found in the literature at normal atmospheric temperatures (20–25 °C) tend to be always higher. This is shown in table 2 for the same materials.

These results might be explained in terms of *kρc* increasing with temperature and by overestimating the *T*_ig_ with then influences *h* in eq (15). These variations in *kρc* and *T*_ig_ should be considered acceptable for assessing ignition and subsequently flame spread when the empirical procedure is compared to the difficulty of measuring *T*_ig_ directly especially for complex materials.

## Opposed Flow Flame Spread

Many investigators have studied the theoretical and fundamental aspects of flame spread on a surface in a direction opposite to a directed flow of the environment [5–10]. This is generally referred to as opposed flow flame spread. The opposed flow may be induced by the spreading flame itself due to buoyancy effects. This would be the case for downward or lateral spread on a wall or horizontal axisymmetric spread on a floor. The natural convection velocities should not vary greatly for small fire conditions and for typical flame temperatures can be estimated at O(10) cm/s [6].

The solution derived by deRis [6] under steady conditions with consideration of the gas phase and solid fuel phase in two dimensions can be given as

$$V_{p}=V_{g}\frac{{\left(k\rho c\right)}_{g}}{k\rho c}{\left(\frac{T_{f}-T_{\mathrm{ig}}}{T_{\mathrm{ig}}-T_{s}}\right)}^{2}.$$ (16)

Here *V_s_* is the opposed flow velocity, *T*_f_ is the flame temperature, and the subscript g denotes properties of the gas phase. Recently Wichman [10] has developed an alternative model which has included effects of finite kinetics in the gas phase. This analysis changes the exponent in eq (16) from 2 to 2.5 and primarily modifies the value of *V*_p_ by a function of a Damköhler number which brings into play needed kinetic data for the fuel. Roughly, if chemical kinetic effects are unimportant then eq (16) is adequate, but if kinetics are important then the actual flame spread speed is lower than that given by eq (16). These results have been shown experimentally for thick (PMMA) and thin (paper) fuels over a wide range of flows (*V*_g_), gravitational fields, and oxygen concentrations [5,7,9,10].

The problem with applying eq (16) in general is the ability to determine the flame temperature for a complex material experiencing opposed flow flame spread. In comparing eq (16) with eq (10) we see that the numerator suggests another “property” that could be sought for specific conditions of opposed flow spread. Here we consider lateral flame spread on a wall in the apparatus illustrated in figure 2 [1–3]. By applying a variable decreasing external radiant heat flux with distance from the ignitor, the flame will spread according to the local surface temperature (*T_s_*). The data can ultimately be correlated by the relationship:

$$V_{p}=\frac{\Phi }{k\rho c\;{\left(T_{\mathrm{ig}}-T_{s}\right)}^{2}},$$ (17)

where Φ is now a new material flame spread “property.” It should be a constant for a particular material under conditions of a fixed *V*_g_ and a fixed ambient oxygen concentration. Thus under natural convection conditions in air we developed values for Φ [2,3]. The results for lateral spread were also found in agreement for downward spread and horizontal axisymmetric spread [4] indicating the opposed flow velocities were similar. This suggests that Φ values derived under lateral spread could also apply to downward (provided melting is not significant) or horizontal spread under natural convection conditions.

If one accepts eq (16) as correct where *T*_f_ is the actual flame temperature, then this value of *T*_f_ can be computed from the data for Φ. For the orientation of lateral spread apparatus if can be argued that natural convection prevails and *V*_g_ is weakly dependent on material through *T*_f_. Selecting *T*_f_ = 2080 °C for purposes of estimating *V*_g_, gives 0.11 m/s [3,6]. Therefore, from eqs (16) and (17)

$$T_{f}=T_{\mathrm{ig}}+\left(\frac{\Phi }{V_{g}{\left(k\rho c\right)}_{g}}\right){\;}^{1/2}.$$ (18)

In order to estimate *T*_f_, (*kρc*)_g_ is taken for air at normal temperature to be 3.3×10^−5^ (kW/m^2^-K)^2^s, and *V*_g_ is taken as 0.11 m/s for natural convection conditions.

The procedure for determining Φ requires several steps. Equation (17) can be rewritten making use of eqs (11)–(15). This results in

$$V_{p}{\left(t\right)}^{-1/2}=\left(\frac{h^{2}\Phi }{k\rho c}\right){\;}^{-1/2}\;\left[{\dot{q}}_{0,\mathrm{ig}}^{\prime \prime }-{\dot{q}}_{e}^{\prime \prime }F\left(t\right)\right].$$ (19)

Here *t* refers to the time the pyrolysis front is at a position *x* and is the time over which ${\dot{q}}_{e}^{\prime \prime }$ has acted at that same position. The empirically derived results from ignition, *F*(*t*), allows us to account for the varying surface temperature over distance *x* and time *t.* This technique has been successful in correlating a wide range of materials over a heat flux range of 0.2 to 5 W/cm^2^ with a wide range of heating times as well [3]. Some illustrative results are shown in figures 5a and 5b. The velocity was determined by analyzing the record of pyrolysis front as a function of time. The lines have been drawn by weighing the data points over the center of the data. This is done for two reasons. *V*_p_ is not accurately determined as ${\dot{q}}_{e}^{\prime \prime }F\left(t\right)$ approaches the intercept ${\dot{q}}_{0,\mathrm{ig}}^{\prime \prime }$; and at the other extreme, extinction effects tend to cause some scatter and curvature in the results. It is interesting to note that this correlation provides a way to determine ${\dot{q}}_{0,\mathrm{ig}}^{\prime \prime }$ independent of the ignition procedure of bracketing. Results for these two materials are summarized in table 3. Generally results for Φ have ranged from approximately 1 to 15 (kW)^2^/m^3^ whereas a value of O(10)(kW)^2^/m^3^ could be estimated from eq (16) using a theoretical flame temperature and a characteristic velocity for natural convection of approximately 10 cm/s. Also shown in table 3 are minimum temperatures for spread (*T*_s,min_) below which no propagation is observed. This cannot be explained theoretically, but must be governed by heat losses and chemical kinetic effects as the surface temperature decreases. The flame spread correlations of figures 5a and 5b show this lower limit for ${\dot{q}}_{e}^{\prime \prime }F\left(t\right)$ from which a corresponding temperature can be computed from eqs (11) and (14), i.e.

$$T_{s,\min}-T_{\infty }=\frac{1}{h}{\left[{\dot{q}}_{e}^{\prime \prime }F\left(t\right)\right]}_{\;\text{lower}\;\text{limit},}$$ (20)

and where *h* is evaluated at *T*_s, min_ by eq (13). Values of *T*_s, min_ range widely. For example, PMMA will allow lateral spread for normal ambient temperatures, while for Douglas fir particle board *T*_s, min_ = 275 °C, and for a fire retarded plywood *T*_s, min_ = *T*_ig_ = 620 °C so that no lateral flame spread was found to be possible.

It is interesting to examine the flame temperature values computed from eq (18). These must be interpreted as not precise flame temperature values, but as approximate indications of the effective flame temperature. The treated wool carpet compared to the untreated version in table 3 suggests that the biggest impact of the “treatment” is in the gas phase on *T*_f_ since the ignition temperature and other properties are not essentially affected. Thus, the test data offer the potential for discerning between gas-phase and solid-phase reductions in flame spread mechanisms for a given material and its additives. This is speculative, but perhaps worthy of further study.

## Upward Flame Spread

Upward flame spread is a particular form of wind-aided flame spread in which the flow velocity in the direction of flame spread is induced by the buoyancy effect of the flame itself. Limited results exist for upward flame spread. Generally, it is found that *V*_p_ is proportional to (*x*_p_−*x*_b_)^n^ where *n* tends to vary from 0.5 to 1. Furthermore the burnout front (*x*_b_) must also be determined. Some examples in the literature give *n* = 0.5 to 0.7 for turbulent conditions over thin textile materials [11], *n* = 3/4 for the thin case and *n* = 1/2 for the thick fuel case both under laminar conditions [12], and for thick PMMA under turbulent conditions *n* was approximately equal to 1 [13].

The theoretical model given by eq (10) has been developed by an alternative analysis by Sibulkin and Kim [14] and has been employed in some recent studies [15–17]. This result lends itself to application to materials particularly if we accept the results already determined for *kρc* and *T*_ig_. Hence we need to seek relationships for ${\dot{q}}_{f}^{\prime \prime }$ and *x*_f_−*x*_p_ in terms of material properties. Some progress has been made in this respect.

The flame heights *x*_f_ have been measured for line diffusion flames of methane and for irradiated wall mounted materials contiguous to a vertical wall. These wall flame heights have been shown under normal ambient air conditions to only depend on the energy release rate per unit wall width for the particular fuel $\left({\dot{Q}}_{b}^{\prime }\right)$ [15,17,18]. The results developed by Delichatsios [18] for turbulent conditions is given below:

$$x_{f}=4.65\left(\frac{{\dot{Q}}_{b}^{\prime }}{C_{p}T_{\infty }\rho_{\infty }\sqrt{g}}\right){\;}^{2/3},$$ (21)

where *c_p_*, *T*_∞_, *ρ*_∞_ are for normal air and *g* is the acceleration due to gravity. All of the fuel chemical properties are contained in ${\dot{Q}}_{b}^{\prime }$. In general, under spreading conditions where burnout is occurring, *x*_f_ in eq (21) should be replaced by *x*_f_−*x*_b_. For (*x*_f_−*x*_b_) < 10 cm the flow is likely to be laminar and eq (21) will not apply, but clearly it will be most applicable to realistic conditions except at incipient spread.

For conditions of constant flame heating ${\dot{q}}_{f}^{\prime \prime }$ over *x*_p_ to *x*_f_ and zero for *x >x*_f_, we can write

$$V_{p}=\frac{x_{f}-x_{p}}{t_{f}},$$ (22)

where $t_{f}=\frac{\pi \;k\rho c{\left(T_{\mathrm{ig}}-T_{s}\right)}^{2}}{4{\left({\dot{q}}_{f}^{\prime \prime }\right)}^{2}}$is a characteristic flame spread time. It was shown by Saito et al. [16] that the application of a modified eq (21) with eq (22) leads to an accelerating result for *x*_p_ provided

$$t_{f}<\frac{\pi (K{\dot{Q}}_{b}^{\prime \prime }{)}^{2}}{4}t_{b},$$ (23)

where $x_{f}-x_{b}=K\;{\dot{Q}}_{b}^{\prime }$is the linear form of eq (21) and *t*_b_ is a characteristic burning time associated with a nominal average energy release rate per unit area ${\dot{Q}}_{b}^{\prime }$ taken as constant over this time. Hence if the energy release rate per unit area is too small or the burning time is too small, eq (23) will not be satisfied and the spread will instead decelerate and terminate. This behavior is independent of the ignition source that got the process started. Although ${\dot{Q}}_{b}^{\prime }$ cannot be regarded as properties, such data are available for at least small samples in combustion calorimeters under conditions of external heating [19]. It is well known that most thermally thick materials will not burn in air unless they receive heat from an external source, e.g., radiant panel. In addition, upward spread will not occur and be sustained (accelerate) unless *t*_f_ satisfies a relationship of the form of eq (23) which depends directly on *t*_b_ and ${\dot{Q}}_{b}^{\prime \prime }$.

**Figure 6:**
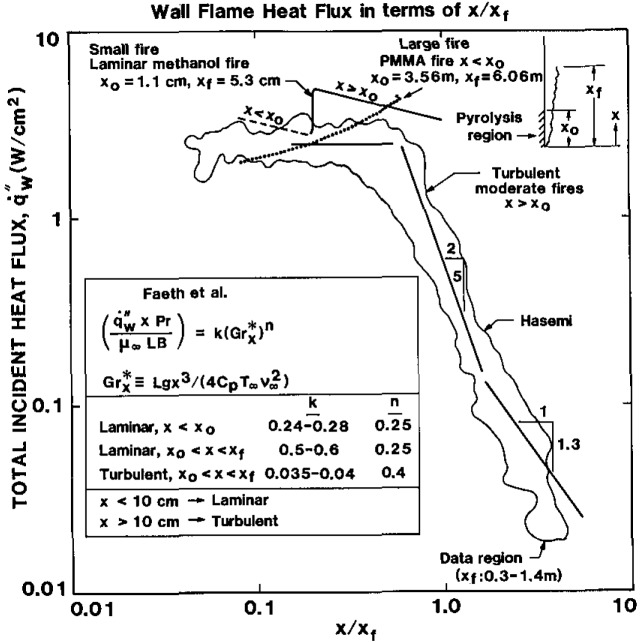
Flame heat flux distribution for wall fires.

Let us now examine the quantity *t*_f_. Presuming we have developed the properties *kρc* and *T*_ig_, we now need to know ${\dot{q}}_{f}^{\prime \prime }$. From figure 4, the flaming ignition in comparison with the pure radiative ignition suggests a level of the flame heat flux to be equivalent to roughly 2 to 3 W/cm^2^. This appears to be independent of the size of the wall flame at least from 20 to 80 kW/m. Indeed this has been the case for wall flames measured for line fuel sources of methane and propane [17,20], a wide variety of materials 0.3 m high irradiated from 1.5 to 3.7 W/cm^2^ [15], and liquid fuel soaked wicks [21]. All of these tend to suggest a universal result independent of the fuel in a plot of ${\dot{q}}_{f}^{\prime \prime }$ with *x*/*x*_f_. A compilation of these results is shown in figure 6. This is the flux above the pyrolysis zone for a burning material. It is striking that the flux in the flame region can be estimated as 2.5±0.5 W/cm^2^ and falls rapidly at *x*>*x*_f_.

These data apply for 0.3⩽*x*_f_⩽1.4 m. We expect for larger flame heights or very sooty fuels that flame radiation effects may distort these results. However over the range of conditions tested it appears that some compensation of radiation and convection is being accommodated to make ${\dot{q}}_{f}^{\prime \prime }$ nearly constant over the flame region. It is interesting to note that the flame heat transfer in the pyrolysis region of a 3.56 m high PMMA wall fire leads to a heat flux of as high as 4.4 W/cm^2^ [22]. Whereas a laminar flame could yield a maximum of 5 W/cm^2^ just outside the pyrolysis zone. (This was calculated from the work of Faeth and co-workers [21].) Hence a laminar wall flame would propagate more easily than a turbulent flame, and a large flame would enhance propagation due to an increase in flame radiation

It is interesting to examine the characteristics of two materials: PMMA and particle board as described in tables 1 and 2. Let us assume that the flame heat flux in the pyrolysis wall region is the same as the value just above, namely 25 kW/m^2^. Also let us consider steady burning so that the burning rate per unit area can be estimated by

$${\dot{m}}_{b}^{\prime \prime }=\frac{{\dot{q}}_{f}^{\prime \prime }+{\dot{q}}_{e}^{\prime \prime }-{\dot{q}}_{0,\mathrm{ig}}^{\prime \prime }}{L},$$ (24)

where *L* is the heat of gasification, and the rate of energy release per unit area is

$${\dot{Q}}_{b}^{\prime \prime }={\dot{m}}_{b}^{\prime \prime }\;\Delta H,$$ (25)

where Δ*H* is the heat of combustion. For the case of ${\dot{q}}_{e}^{\prime \prime }=0$ and *T*_s_ = 20 °C we estimate whether flame propagation is possible. This is shown in table 3 where the property values were taken from tables 1 and 2 and reference [15]. The burning times *t*_b_ were taken from our experience with the specific material examined [15]. The value *K* was based on a linearized flame height relationship; *K* ≈ 0.01 m^2^/kW for *x*_f_ = 1 m.

The results from table 4 suggest the wood material will not propagate while the PMMA easily leads to an accelerating flame spread. These results have been confirmed by experiments on these same materials [16]. Indeed, only by increasing *T*_s_ with external heating has led to reducing *t*_f_ and propagation for the wood [23]. Clearly this analysis has been highly approximate, and its refinement should be based on experimental results. Recent experimental results for this particle board have shown that it is possible to have flame spread under external heating conditions as low as ${\dot{q}}_{e}^{\prime \prime }=0.42\;W/{\mathrm{cm}}^{2}$ and corresponding *T*_s_ values of roughly 100 °C. This is lower than the analysis in table 4 would suggest. But, at least, the trend and framework given by eq (22) appears valid.

## Conclusions

A summary of work has been presented which attempts to develop a procedure for determining property data for flame spread. The procedure appears to work for a wide variety of materials for the case of lateral flame spread on vertical walls these effective properties are listed as follows:

1. *kρc*, which is a measure of the rate of temperature rise for a material;
2. *T*_ig_, which is a measure of the temperature at which the material is volatized sufficiently to form a flammable mixture in air; and
3. Φ, which is a measure of the flame heat transfer under conditions of opposed flow natural convection in air.

For the case of upward flame spread on a vertical surface, some of these same properties apply. But now the flame heat transfer is a function of time which depends on the energy release rate and burning time of the material. Moreover not just the rate of upward flame spread is governed by these additional characteristics, but they also control whether spread will accelerate and be sustained. Some results have been discussed to illustrate this aspect of upward flame spread. More study is needed to generalize these results into a complete measurable set of properties for predicting upward spread.

## Figures and Tables

**Figure 3a:**
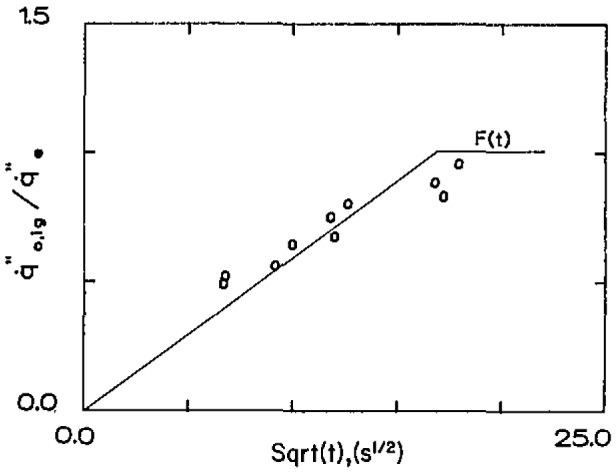
Pilot ignition results under radiative heating—polycarbonate, ${\dot{q}}_{0,\mathrm{ig}}^{\prime \prime }=3.0\;W/{\mathrm{cm}}^{2}$.

**Figure 3b:**
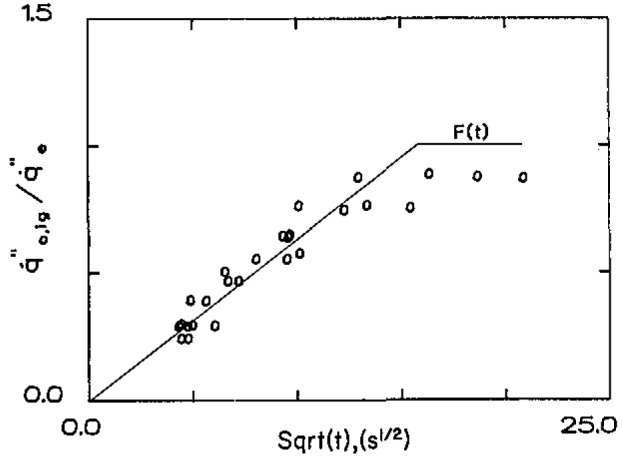
Pilot ignition results under radiative heating—carpet, ${\dot{q}}_{0,\mathrm{ig}}^{\prime \prime }=1.8\;W/{\mathrm{cm}}^{2}$.

**Figure 5a:**
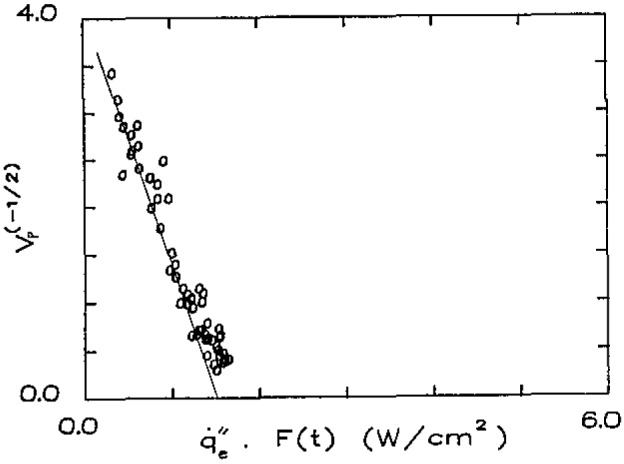
Correlations of lateral flame spread—asphalt shingle.

**Figure 5b:**
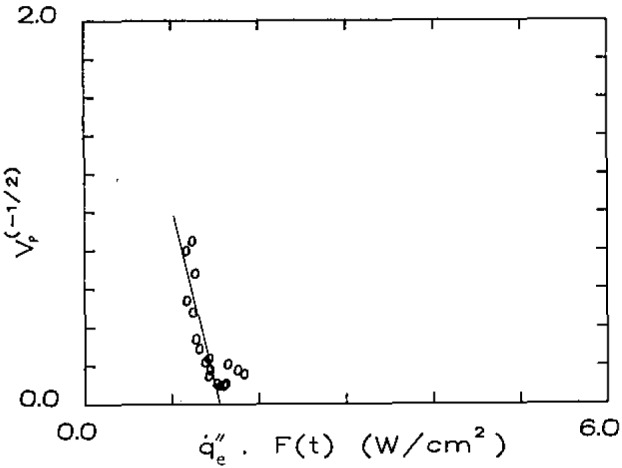
Correlations of lateral flame spread—wool carpet #2 (untreated).

**Table 1:** Comparison of measured and derived ignition temperatures under radiative heating

Material	Radiative heating (W/cm^2^)	Measured, *T*_ig_ (°C)	Derived, *T*_ig_ (°C)
Particle board	2.0–6.3	290–380	382
PMMA	1.6–4.6	285–330	378

**Table 2:** Comparison of derived and literature values of *kρc*

Material	Derived value	Literature value
		(25 °C)
	(kW/m^2^K)^2^s	(kW/m^2^K)^2^s
PMMA	1.02	0.66
Particle board	0.93	0.14

**Table 3:** Illustrative results for lateral flame spread

Material	${\dot{q}}_{0,\mathrm{ig}}^{\prime \prime }$ (ignition data) W/cm^2^	*T*_ig_ °C	${\dot{q}}_{0,\mathrm{ig}}^{\prime \prime }$ (spread data) W/cm^2^	Φ (kW)^2^/m^3^	*T*_f_ °C	*T*_s, min_ °C
Asphalt shingle	1.5	378	1.6	5.4	1590	140
Wool carpet #2 (untreated)	2.0	435	1.6	7.3	1850	335
Wool carpet #2 (treated)	2.2	455	1.6	0.89	950	365
PMMA (G)	1.5	378	1.6	14.4	2370	<90

**Table 4:** Examination of upward spread potential

Material	*t*_b_ (s)	*L* (kJ/g)	Δ*H* (kJ/g)	${\dot{Q}}_{b}^{\prime \prime }$ (kW/m^2^)	*t*_f_ (s)	$\left(\pi /4\right){\left(K{\dot{Q}}_{b}^{\prime \prime }\right)}^{2}t_{b}$ (s)
Particle board	400.	4.5	12.	23.	153.	16.
PMMA	1400.	1.8	26.	116.	161.	1468.
